# Noninvasive in-vivo tracing and imaging of transplanted stem cells for liver regeneration

**DOI:** 10.1186/s13287-016-0396-y

**Published:** 2016-09-23

**Authors:** Panpan Cen, Jiajia Chen, Chenxia Hu, Linxiao Fan, Jie Wang, Lanjuan Li

**Affiliations:** Collaborative Innovation Center for Diagnosis and Treatment of Infectious Diseases; State Key Laboratory for Diagnosis and Treatment of Infectious Diseases, School of Medicine; First Affiliated Hospital; Zhejiang University, Hangzhou, 310006 China

**Keywords:** Stem cells, Stem cell therapy, In-vivo imaging, Labeling, Optical imaging, Radionuclides, Super paramagnetic iron oxide, Reporter genes, Liver regeneration

## Abstract

Terminal liver disease is a major cause of death globally. The only ultimate therapeutic approach is orthotopic liver transplant. Because of the innate defects of organ transplantation, stem cell-based therapy has emerged as an effective alternative, based on the capacity of stem cells for multilineage differentiation and their homing to injured sites. However, the disease etiology, cell type, timing of cellular graft, therapeutic dose, delivery route, and choice of endpoints have varied between studies, leading to different, even divergent, results. In-vivo cell imaging could therefore help us better understand the fate and behaviors of stem cells to optimize cell-based therapy for liver regeneration. The primary imaging techniques in preclinical or clinical studies have consisted of optical imaging, magnetic resonance imaging, radionuclide imaging, reporter gene imaging, and Y chromosome-based fluorescence in-situ hybridization imaging. More attention has been focused on developing new or modified imaging methods for longitudinal and high-efficiency tracing. Herein, we provide a descriptive overview of imaging modalities and discuss recent advances in the field of molecular imaging of intrahepatic stem cell grafts.

## Background

Liver dysfunction is a serious healthcare problem worldwide that can progress to fulminant or chronic liver failure, and eventually deteriorate into end-stage liver disease. Currently, the only ultimate therapeutic approach for these diseases is orthotopic liver transplant (OLT). Nevertheless, the potential benefits are extraordinarily hindered by the major characteristics of organ scarcity, surgical intervention, postoperative complication, and life-long immunosuppressive medication, which have urgently facilitated the exploration of novel strategies to promote hepatic self-rehabilitation ability and reverse the pernicious process.

Early observations that stem cells derived from somatic cells, bone marrow, and embryonic cells exhibit the capacity of multipotential differentiation and self-renewal in vitro led to the proposal that they might migrate to the injured sites driven by environmental triggers and partly substitute the function of hepatocytes. Thus, over the last several years, stem cell-based therapy has emerged as a possible alternative, revolutionizing the treatment of liver regeneration or enabling patients to buy time before liver transplantation [1–4]. By homing to damaged tissues, stem cells contribute to alleviating the liver dysfunction. However, the potential mechanisms involved are not yet completely understood. Moreover, the disease etiology, cell type, timing of cellular graft, therapeutic dose, delivery route, and choice of endpoints have varied between study groups, leading to different, even divergent, treatment outcomes. Optimizing stem cell-based therapies will therefore require a better understanding of the cellular viability, biodistribution, differentiation capacity, and long-term fate after engraftment, with imaging techniques playing a pivotal role. Successful implementation of proper cell labeling enables noninvasive monitoring for the in-vivo tracing of cellular biology, and provides some clues for stem cell therapies.

An ideal imaging technique should most of all be biocompatible; that is, with low toxicity to both the labeled cells and the host. Additionally, the imaging technique should allow the relatively long-term visualization of infused cells with high temporary and spatial resolution, and meanwhile be available for histological or functional analysis. In addition, it is vital that the labeling agent or marker should be highly specific to original cells, passed to all progenies, and not transfected to nontarget cells [5, 6]. To the best of our knowledge, there is no single imaging method that satisfies all of the ideal conditions. However, we can combine multiple imaging strategies to achieve optimal imaging sensitivity, resolution, and time for follow-up.

Currently, in-vivo cell tracing in liver tissues is performed by direct labeling techniques or reporter gene labeling. Direct labeling is the most straightforward method, introducing imaging-detectable probes into target cells before implantation, including dye-mediated optical imaging, magnetic resonance imaging (MRI), and radionuclide imaging. A few researchers have also utilized Y chromosome-based fluorescence in-situ hybridization (FISH) imaging. In this review, we discuss the advantages and disadvantages of these imaging modalities and provide an overview of the recent advances in the domain of molecular imaging of intrahepatic stem cell grafts.

### Dye-mediated optical imaging

Fluorescent probe labeling was developed for direct-viewing detection during in-vivo applications of cellular visualization and monitoring. This method provides the basis of other subsequently developed tracer techniques. Connection of fluorophores to labeled stem cells would allow detection of migrated stem cells by postmortem liver tissues using a fluorescent microscope. Thus far, a variety of fluorescent dye has been utilized, which can reversibly or irreversibly bind to the cell nucleus (e.g., 4′,6-diamidino-2-phenylindole (DAPI), bis-benzimide (Hoechst), 5-bromo-2′-deoxyuridine (BrdU)) or the cell membrane (e.g., PKH26, 1,1-dioctadecyl-3,3,3,3-tetramethylindotricarbocyanine iodide (DiR)), or locate in the nucleus, membrane, and cytoplasm (e.g., carboxyfluorescein succinimidyl amino ester (CFSE)).

To date, a large number of animal studies involving different liver disease models have been promoted as a result of preclinical data in favor of fluorescent dyes for stem cell in-vivo tracing (Table 1). For example, the membrane lipophilic dyes DiR [7] and CM-DiI [8] have effectively labeled bone marrow-derived mesenchymal stem cells (BMSCs) and showed no impairment of cytomophology and cell viability. PKH26 is a safe fluorescent marker with good biocompatibility, although a recent study has indicated that PKH26 labeling was not specific to the transplanted cells [9]. Moreover, the groups of both Ikeda et al. [10] and Zhan et al. [11] have used fluorescent PKH26 dye to demonstrate the capacity of stem cells to differentiate into hepatocytes in pathologic hepatic environments and express liver-specific markers Alb, CK8, and CK18.

**Table 1:** Intrahepatic animal stem cell tracking studies with dye-mediated optical imaging

Study	Species (*n*)	Animal model	Cell type	Agent	Delivery/number of cells infused	Study observations
Ma, 2014 [7]	Mice (75)	CCl4-induced ALF model	Xenogeneic BMSCs	DiR	Caudal vein/10^6^	At 5 days after transplantation, a strong fluorescent signal from labeled CXCR4 MSCs was almost distributed in the liver, whereas in the null group the liver and spleen transmitted nearly the same signal intensity
Sun et al., 2013 [8]	Rats (18)	CBDL-induced obstructive liver disease model	Allogeneic BMSCs	CM-DiI	Intrasplenic injection/10^6^	Detection of fluorescence-labeled cells after 1 week The labeling procedure did not impair cytomophology The fluorescent images showed that the IOD was significantly larger in experiment group, and the signals presented unevenly distribution in the fibrous liver tissue
Ikeda et al., 2008 [10]/Zhan et al., 2006 [11]	Rats (7/unknown)	CCl4-induced liver injury model	Allogeneic TGPCs/HSC	PKH26	Portal vein/10^7^	The red fluorescent cells demonstrated the capacity of stem cells to migrate, proliferate, and differentiate in pathologic hepatic environments after engraftment
Li et al., 2013 [12]	Mice (14)	MHCC97-H-induced HCC model	Xenogeneic BMSCs	RFP, GFP, BrdU, DAPI	Caudal vein/10^5^	Luminescent binucleated cells were seldom observed both in vitro and in vivo for a long-term follow-up period After 4 days, most BMSCs grafted to the tumor focus, and after 20 days, labeled MSCs almost accumulated in the tumor stroma
Ezzat et al., 2012 [13]	Mice (40)	APAP-induced ALF model	Allogeneic ESCs	DiR, GFP	Intrasplenic injection/10^6^	DiR-labeled cells accumulated in the spleen within 30 min, moved to the liver at 3 hours, disseminated to almost all regions of the liver at 24 hours, and faded at 72 hours GFP-positive cells were found under the liver capsule and were still detected after 2 weeks
Akham et al., 2015 [15]	Rats (6)	PHx-induced liver injury model	Allogeneic BMSCs	CPN	Caudal vein/10^6^	Postmortem liver tissue showed the presence of luminescent cells at the injury lesions and retained there The labeling process did not impair the marker expression, multilineage differentiation ability, or cell viability
Yukawa et al., 2012 [16]	Mice (18)	CCl4-induced ALF model	Allogeneic AD-MSCs	QDs	Caudal vein/10^6^	Within 10 min, 70 % of fluorescent signal retained in the lungs and 30 % of signals came from the liver when AD-MSCs were transplanted with heparin After 1 day, the accumulation rate decreased to 10 % in both organs and maintained for at least 2 days

*CCl4* carbon tetrachloride, *BMSC* bone marrow-derived mesenchymal stem cell, *ALF* acute liver failure; *CXCR4* chemokine CXC receptor 4, *CBDL* common bile duct ligation, *IOD* integral optical density, *HSC* hematopoietic stem cell, *TGPC* tooth germ progenitor cell, *HCC* hepatocellular carcinoma, *GFP* green fluorescent protein, *RFP* red fluorescent protein, *APAP* acetaminophen, *ESC* embryonic stem cell, *PHx* partial hepatectomy, *CPN* conjugated polymer-based water-dispersible nanoparticles, *AD-MSC* adipose-derived mesenchymal cell, *QD* quantum dot, *DAPI* 4′,6-diamidino-2-phenylindole, *BrdU* 5-bromo-2′-deoxyuridine, *DiR* 1,1-dioctadecyl-3,3,3,3-tetramethylindotricarbocyanine iodide

However, narrow penetration depth and unsatisfactory photostability of traditional fluorescent dyes are the most severe limitations for in-vivo imaging even in rodents, let alone use for human intrahepatic detection. In order to achieve real-time visualization and longitudinal tracking results, dual or multiple labeling [12, 13] and new types of near-infrared fluorescence probes (e.g., quantum dots (QDs) [14], conjugated polymer-based water-dispersible nanoparticles (CPNs) [15]) have been implemented to optimize molecular imaging. QDs consist of inorganic semiconductor nanoparticles, provide a narrow emission spectrum to reduce autogenic fluorescence, allow exact single molecule positioning, and possess superior photostability. Peripheral intravenous delivery of QD-labeled MSCs has been successfully tracked in the liver for at least 2 days [16]. While in cardiovascular application, QD-labeled stem cells were reported to maintain in the heart for at least 8 weeks [17]. The particular optical advantages make QDs seem to be promising candidates for long-term in-vivo imaging for liver regeneration.

Proper cell labeling is essential to better understand cellular biology and provide clues for stem cell therapies. In most of the cell tracking animal studies, stem cells were well distributed in the liver after transplantation for a relatively long-term period through the spleen or portal vein. This may indicate that peripheral intravenous injection is not the optimal route of cell therapy for renewal of liver function owing to the major cell retention outside the liver. In addition, no studies have shown impaired cell viability and differentiation ability. Thus, because of their relative safety, high efficiency, low cost, and ease of use, fluorescent probes have gained extensive application for both in-vivo and in-vitro experiments. Fluorescence probes provide high sensitivity and allow for multiple labeling according to their different optical spectrum characteristics [18]. They emit varying intensities and colors of fluorescence simultaneously or successively to satisfy the different requirements of tracing. However, poor spatial resolution, shallow penetration, and inevitable light decay along with cell division have largely restricted this imaging strategy to visualizing deep anatomy such as hepatic sinusoid without invasive manipulation. New types of nanoparticle probes (e.g., QDs) have emerged to circumvent some of the problems, but the high doses may increase nontarget binding and the safety issue still requires attention [19]. Therefore, dye-mediated optical techniques are currently suitable for short-term tracking and preclinical application. We still have to face great challenges before they can be pushed into clinical practice.

### Magnetic resonance imaging

With the development of molecular imaging, MRI has emerged as a noninvasive and sensitive technique for longitudinal tracing of the distribution, retention, homing, and differentiation of transplanted progenitor cells in intact living organisms. Since MRI combines high spatial (25–100 μm) and soft-tissue resolution with free selection of the imaging plane [18], MRI techniques have been widely applied in animal research for in-vivo tracing of transplanted stem cells (Table 2).

**Table 2:** Intrahepatic animal stem cell tracking studies with MRI

Study	Species (*n*)	Animal model	Cell type	Agent	Delivery/number of cells infused	Study observations
Pang et al., 2015 [22]	Rats (18)	CCl4-induced liver fibrosis model	Allogeneic BMSCs	PEG-g-PEI-SPIO	Mesenteric vein/10^6^	Detection of modified cells for up to 2 weeks post transplantation Labeled cells were still present in the liver intralobular parenchyma after 2 weeks The labeling process displayed good biocompatibility
Zhao et al., 2014 [23]	Mice (12)	CCl4-induced liver injury model	Xenogeneic AD-MSCs	SPIO	Splenic vein/10^7^	Hypointense MRI images were detected until 7 days The attenuation of MRI signals mainly arose from excretion of SPIO Fluorescence and PB staining showed that the SPIO particles were still inside the stem cells The location of AD-MSC accumulation was well integrated with the liver injury focus
Chen et al., 2012 [25]	Rats (40)	PHx-induced liver injury model	Allogeneic BMSCs	SPIO	Directly intrahepatic into residual lobe/10^6^	An oval hypointense area at injection sites was visible within 2 weeks by MRI, while the signal intensity decreased with time PB stain showed the presence of Feridex-labeled cells in the liver sinusoid
Wang et al., 2014 [26]	Mice (12)	CCl4-induced liver injury model	Allogeneic EPCs	SPIO	Caudal vein/10^6^	Detection of grafted cells after 8 days PB stain revealed SPION containing stem cells accumulated in the liver parenchyma, particularly along sinusoids and portal areas
Bos et al., 2004 [27]	Rats (4)	CCl4-induced ALF model	Allogeneic BMSCs	SPIO	Portal vein/10^6^	Detection of cells up to 12 days Signal intensity loss of MRI appeared a granular pattern Matching areas stained positive for PB and CD90 antigen of postmortem liver tissue showed the SPIO particles were retained in the originally labeled cells
Cai et al., 2008 [28]	Rats (30)	CCl4-induced ALF model	Allogeneic BMSCs	SPIO	Hepatic artery/10^6^	Hypointense MRI images faded over time and were detected within 7 days Cell viability was not impaired by labeling procedure for up to 4 weeks PB staining and DAPI-stained blue fluorescent nuclei showed the presence of original iron particles containing cells
Zhou et al., 2010 [29]	Rats (18)	CCl4-induced liver fibrosis model	Allogeneic BMSCs	SPIO	Mesenteric vein/10^6^	Detection of grafted cells for 12 days in BMSC-labeled group, but for only 3 days in cell-free SPIO group PB staining showed the presence of originally labeled cells in the portal region at 3 days, and mainly in the injured areas of intralobular parenchyma at 15 days
Kim et al., 2010 [31]	Rats (14)	DMN-induced liver fibrosis model	Xenogeneic BMSCs	MNP SPIO	Intrasplenic injection/10^6^	Detection of transplanted cells after 14 days The decrease of MRI signal intensity was more obvious in MNP-tagged group. Masson trichrome staining and autofluorescent images of MNP-tagged cells showed most stem cells migrated to the fibrous septa
Ju et al., 2007 [32]	Rats (12)	CCl4-induced liver cirrhosis model	Allogeneic BMSCs	SPIO	Splenic vein/10^6^	Detection of injected cells for up to 2 weeks No visible blue particles were found in unlabeled cells after PB staining Grafted cells were mainly distributed in periportal and injured areas

*CCl4* carbon tetrachloride, *BMSC* bone marrow-derived mesenchymal stem cell, *PEG-g-PEI-SPIO* superparamagnetic iron oxide nanoparticles coated with polyethylene glycol-grafted polyethylenimine, *AD-MSC* adipose-derived mesenchymal cell, *SPIO* superparamagnetic iron oxides, *PB* Prussian blue, *DAPI* 4′,6-diamidino-2-phenylindole, *PHx* partial hepatectomy; *MRI* magnetic resonance imaging; *EPC* endothelial progenitor cell, *ALF* acute liver failure; *DMN* dimethylnitrosamine; *MNP* fluorescent magnetic nanoparticle

Currently, gadolinium chelates (Gd^3+^-DTPA) and superparamagnetic iron oxides (SPIO) are the most frequently used imaging agents for in-vivo detection with MRI. Lanthanide gadolinium, known as T1 contrast agent, produces an enhanced intensity zone on T1-weighted MRI images, while SPIO nanoparticles generate hypointense areas with the T2 modality. Superparamagnetic agents are usually composed of a crystal core, typically 3–5 nm in mean diameter. Because of their good biocompatibility and high relaxivity, SPIO nanoparticles have been considered to be a preferred MRI agent tracer for cell labeling. Moreover, these contrast agents have been FDA approved as a liver agent for clinical use (SPIO: Feridex, USA; Endorem, Europe) [20].

Much effort has been made to optimize the internalization process for high-efficiency cellular magnetic labeling of stem cells, since they lack the ability of phagocytosis. So far, the most commonly used method is magnetofection, which requires transfection agents (most are cationics), such as lipofectamine, poly-l-lysine, dextran, and protamine sulfate, to envelop SPIO nanoparticles by electrostatic interactions and pass through the cell membrane [21]. Nonionic dendrimer-coated SPIO particles have also been reported to track BMSCs in rats with liver fibrosis for 2 weeks [22]. One disadvantage of current commercially available transfection agents is the long incubation periods resulting in the risk of altering their properties, although no research has shown that the transduction procedures influenced the viability or multilineage differentiation potential of stem cells in appropriate media and activated inflammatory response [23]. To achieve high uptake rates and efficient labeling, SPIO were reported to have been derivatized with HIV-Tat protein-derived peptide sequences for in-vivo tracing [24]. In numerous preclinical studies, various stem cell lines have been effectively labeled by magnetofection and tracked in the liver for several weeks using MRI in vivo [25–29]. The hypointense MRI was well integrated with the focus of liver injury, indicating that transplanted stem cells have the ability to home to where they are most needed. However, these agents are not yet clinically approved, which may raise safety concerns with patients for their potential toxicity. Another strategy to induce intracytoplasmatic magnetic labeling is magnetoelectroporation (MEP), a more rapid and efficient method to mediate endocytosis of SPIO [30]. Based on the mechanism of lower voltage pulses, this technology does not require transfection agents, which to some extent circumvents a major barrier for clinical application. Although there have been no animal or clinical studies using magnetoelectroporation for labeling of intrahepatic stem cell grafts, further explorations can be prompted. In addition, fluorescent magnetic nanoparticle (MNP), a new commercially tagging material, was applied in a liver cirrhosis rat model [31]. MNP obtains the strong points of both magnetism and optics, and no transfection agents are needed for cell labeling, thus also efficiently avoiding potential threats for the clinic.

The mechanisms of gradual signal intensity restoration of MRI are not yet fully understood. Several scholars have attributed the signal drop mainly to the excretion of SPIO, which was confirmed by histological examination in the study by Zhao et al. [23], by coincidence capturing the possible excretion route of SPIO and showing that the liver macrophages engulfed cell fragments together with the SPIO agent. Another group showed no Kupffer cells containing blue particles were observable on histological analysis [32]. Thus, the proposed mechanism was that the labeled cells were gradually mobilized out of the liver. Consequently, a major limitation of MRI is that the direct-viewing detection of labeled cells depends on the appearance of SPIO within the cells. It is difficult for us to recognize the iron particles released from labeled cells. False positive results might occur if neighboring hepatocytes engulf the iron oxide [33]. Another disadvantage is that SPIO, as a T2 contrast agent, produces gradually attenuated intensity areas on MRI images during the follow-up period, thus rendering it difficult for quantification, because it is difficult to draw cause–effect conclusions about the signal intensity and cell viability [14]. Finally, regarding clinical application, people with implant cardiac pacemakers or metal foreign bodies are contraindicated.

Despite these concerns, MRI has potential for short-term or long-term tracing of intrahepatic dynamic migration processes of stem cells. MRI can provide three-dimensional imaging with high spatial and temporary resolution, which is well suited for implementing the subtle positioning, such as in the sinusoids, portal, or perihepatic areas, and performing qualitative analysis of stem cell fate and behavior. Several scholars have demonstrated the feasibility of MRI for noninvasive visualization of various stem cells grafted to cardiovascular tissue and the nervous system for clinical trials [34–36]. In the field of liver diseases, the move of regeneration medicine from bench to bedside remains to be undertaken. Novel developments in MRI-visible tagging materials need to be focused on to further achieve this transition.

### Radionuclide imaging

Single-photon emission computed tomography (SPECT) and positron emission tomography (PET) are highly sensitive (10^–11^–10^–12^ mol/l) modalities available for radionuclide imaging, which can separately detect γ-emitting isotopes (e.g., ^111^In, ^131^I, ^99m^Tc) and positron-emitting isotopes (e.g., ^18^F, ^124^I, ^68^Ga) with short half-lives to follow cellular trafficking and biodistribution [18]. PET has a preferable spatial and temporal resolution (~1 cm/second to minute) to SPECT (1–2 cm/minute), but is far inferior to MRI [37]. Like optical labeling methods, stem cells are tagged by direct incubation with the radioactive tracers, which passively penetrate the cell membrane and bind to different intracellular components within a short time [38].

In-vivo radionuclide imaging of infused stem cells labeled with various isotopes has shown promising results for noninvasive tracing in both preclinical and human studies, although the labeling technique has not been widely used for intrahepatic applications. For clinical trials in humans, ^111^In-oxine, ^99m^Tc, and ^18^F-FDG are the most extensively used agents for PET or SPECT imaging. Gholamrezanezhad et al. [39] recruited four patients with uncompensated liver cirrhosis into a study, and the patients underwent intravenous injection of ^111^In-oxine-labeled MSCs with tagging efficiency from 36 to 53 %. SPECT images showed constantly increasing radioactivity in the liver within 10 days post infusion and no unexpected side effects occurred over 1 month of follow-up. In another clinical phase 1 study, the migration of ^99m^Tc-labeled bone marrow-derived mononuclear cells following hepatic arterial transplant into eight patients with liver cirrhosis was identified by scintigraphy [40]. In-vivo imaging after 3 and 24 hours showed 41 % and 32 % of the total radioactivity, respectively, in the liver tissue. Ameliorative liver function by biochemical tests was also observed.

However, some persistent disadvantages cannot be ignored. The short half-lives of radioisotopes, such as ^99m^Tc with 6 hours, ^18^F with 110 minutes, and ^124^I with 4 hours, largely limit the long-term detection of radioactivity, so that only the immediate imaging of cellular behavior can be analyzed. For example, the group of Li et al. [41] provided evidence for the mobilization of ^131^I-labeled induced pluripotent stem cells (iPSCs) after peripheral infusion from their original localization to the liver of mice. Serial monitoring of radioactivity signals by γ-camera revealed that the in-vivo signal intensity suffered a progressive recession at 12 hours post cell transplantation. Additionally, time-dependent cytotoxic effects and ionizing radiation damage to target cells still need to be properly assessed. Because radiotracers may risk being excreted from the originally labeled cells, confusing signals could be generated from free radioactive isotopes, which can lead to inaccurate investigations [42].

In summary, the successful implementation of tracing isotope-labeled stem cells homing to the liver demonstrates the feasibility of radionuclide imaging as a noninvasive tool to monitor cell delivery, biodistribution, and fate for clinical practice. However, the short half-life of radionuclides is the major clinical barrier for long-term tracing. Longer-lived radioisotopes should be explored in the future, also taking into account the biosecurity and practicability.

### Reporter gene labeling

Direct labeling modalities universally suffer from inevitable signal attenuation, accompanied by cellular division over time. To circumvent this limitation, reporter gene imaging was developed to trace the multilineage of progenitor cells in complex systems of interest. The reporter genes are transferred into the genome of target stem cells via viral or nonviral methods, including transfection of expressing plasmid cDNA, transduction with lentiviral/adenoviral/cytomegaloviral vectors, or acquisition from transgenic animals [43]. Once they are incorporated, the reporter genes, which are activated by endogenous or exogenous promoters, encode the overexpression of various reporter proteins, such as fluorophores (green fluorescent protein (GFP)), enzymes (herpes simplex virus type 1 thymidine kinase (HSV-tk), luciferase, β-galactosidase), receptors (dopamine 2 receptor), and transporter proteins (sodium iodide symporter (NIS)) [37]. After the implementation of reporter probes, the generated signals are immediately captured by different imaging devices; for example, optical charge-coupled device (CCD), SPECT, or PET.

Intrahepatic cell tracking using reporter genes has shown a promising outlook, although the technique is currently used in animal studies for some ethical concerns (Table 3). Real-time visualization of the cellular biodistribution in the liver was dynamically assessed by luciferase-based bioluminescence imaging (BLI) [44–46]. Bioluminescence provides excellent sensitivity reaching 10^–15^–10^–17^ mol/l and relatively high throughput, but it is limited by a weak depth of penetration of 1–2 cm with a high absorption rate in hemoglobin [47]. Furthermore, the luminescent cells in damaged liver tissue exhibited a hepatocyte-like morphology and human alpha fetal protein (AFP) expression was detected in corresponding regions, which well documented that liver regeneration was motivated by the homing behavior of stem cells toward environmental cues and their potential hepatogenic differentiation [48]. Another widely used reporter gene is GFP for fluorescent imaging (FLI) [49, 50]. Encouraging results have been found by Song et al. [51], showing that implanted GFP-positive cells could survive in the liver for up to 18 weeks and accounted for approximately 40–50 % of regenerative hepatocytes. However, the limitation of relatively low spatial resolution is similar to that of dye-mediated optical imaging. NIS is a transmembrane glycoprotein mainly expressed in the thyroid glands and mediates the process of iodine intake. Reporter genes for nuclear imaging based on NIS (PET or SPECT) are also available with the advantage of quantitative analysis and potential clinical prospects [52, 53]. Other types of available reporter genes include galactosidase genes (LacZ) for bioluminescence imaging [54] and HSV-tk for PET [55].

**Table 3:** Intrahepatic animal stem cell tracking studies with reporter genes

Study	Species (*n*)	Animal model	Cell type	Reporter genes	Reporter probe	Delivery/number of cells infused	Results
Duan et al., 2007 [45]	Mice (12)	NOD-SCID model	Xenogeneic EPCs	Luc	BLI	Directly intrahepatic into parenchyma/10^5^	Detection of luminescent stem cells for at least 1 week
Boeykens et al., 2013 [46]	Rats (13)	PHx of MCD-induced steatotic liver model	Allogeneic BMSCs	Luc	BLI	Portal vein and tail vein/10^6^	Detection of luminescent stem cells for 24 hours Intraportal cell injection was superior to intravenous cell injection for homing capacity
Di Rocco et al., 2012 [48]	Mice (32)	CCl4-induced ALF model	Autologous AD-MSCs	Fluc	BLI	Intrasplenic injection/10^5^	Luciferase-positive cells were visible for 2 months Labeled cells exhibited a hepatocyte-like morphology and AFP expression was detected at injured sites
Li et al., 2009 [49]	Mice (unknown)	CCl4-induced liver cirrhosis model	Allogeneic BMSCs	EGFP	FLI	Tail vein/10^6^	Labeled cells were detected for up to 6 weeks The presence of double-positive cells for EGFP and α-SMA in the fibrous liver demonstrated the homing ability of BMSCs to damaged tissue and differentiation potential into myofibroblasts
Song et al., 2004 [51]	Mice (unknown)	PHx-induced liver injury model	Autologous LPCs	GFP	FLI	Intrasplenic injection/10^6^	GFP-positive cells could be visualized for up to 18 weeks, and accounted for 40–50 % of regenerative hepatocytes
Knoop et al., 2011 [52]	Mice (47)	Huh7-induced HCC model	Xenogeneic BMSCs	NIS	^123^I (γ-camera) ^124^I (PET)	Tail vein/10^5^	NIS-MSC mediated concentration of iodine radioisotopes was detected in 74 % of tumors with a half-life period of 4 hours ^124^I as reporter probe for PET imaging provided better resolution and sensitivity
Knoop et al., 2013 [53]	Mice (57)	Huh7-induced HCC model	Xenogeneic BMSCs	NIS	^131^I (γ-camera)	Tail vein/10^5^	NIS-MSC mediated concentration of radioisotopes was detected in 67 % of tumors with a half-life period of 3.7 hours
Kanazawa et al., 2011 [54]	Rats (24)	I/R injury model	Allogeneic BMSCs	LacZ Luc	X-gal (microscope) (BLI)	Portal vein/10^6^	BLI and histological findings showed that injected stem cells survived in the remnant liver for up to 168 hours LacZ-positive stem cells were mainly located around the periportal regions

*NOD-SCID* nonobese diabetic–severe combined immunodeficiency disease, *EPC* endothelial progenitor cell, *Luc* luciferase, *BLI* bioluminescence imaging, *PHx* partial hepatectomy, *MCD* methionine/choline-deficient, *BMSC* bone marrow-derived mesenchymal stem cell, *CCl4* carbon tetrachloride, *ALF* acute liver failure, *AD-MSC* adipose-derived mesenchymal cell, *Fluc* firefly luciferase, *AFP* alpha fetal protein, *EGFP* enhanced green fluorescent protein, *FLI* fluorescence imaging, *α-SMA* alpha smooth muscle actin, *LPC* liver progenitor cell, *GFP* green fluorescent protein, *HCC* hepatic cellular cancer, *NIS* sodium iodide symporter, *PET* positron emission tomography, *I/R* ischemia–reperfusion, *LacZ* galactosidase genes

Because gene transduction into the host cells is stable and well tolerated, the signals radiating from reporter genes cannot be easily diffused or diluted with cell division, which is appropriate for long-term in-vivo tracing and monitoring [5]. Since this labeling technique requires molecular manipulations of the stem cells before transplantation, concerns over theoretical risks and feasibility for clinical application must be fully assessed, including: viral vector toxicity from xenogenic materials; the expression stability of reporter genes before migrating to the liver; precise integration into the injured liver as much as possible; hepatic immunogenic responses towards accumulation of reporter gene products; and ethical problems [56, 57].

### Y-chromosome marker

The Y-chromosome marker has been utilized in a few sex-matched studies in which cells isolated from male donors were grafted to female recipients, thus allowing us to track the biological actions of transplanted stem cells via the molecular-cytogenetic FISH technique For example, a clinical cell imaging study was successfully performed in four female patients with confirmed alcoholic hepatitis, who underwent cross-sex stem cell transplantation [58]. FISH for the Y chromosome with immunostaining demonstrated the differentiation ability of BMSCs into hepatic myofibroblasts. Using a Y-chromosome-selective probe, the tracing method avoids the latent problems of viral vector transduction, transgenic labeling, cytotoxic effects, signal decay, or diffusion, making it fairly suitable for the long-term assessment of donor cells in recipients [59]. The technique is also easy to perform with high labeling efficiency, and is barely affected by tissue types and phenotype changes. However, the fatal weakness is that Y-chromosome marker cannot apply to autologous transplantation or female donors.

## Conclusion and perspectives

**Fig. 1:**
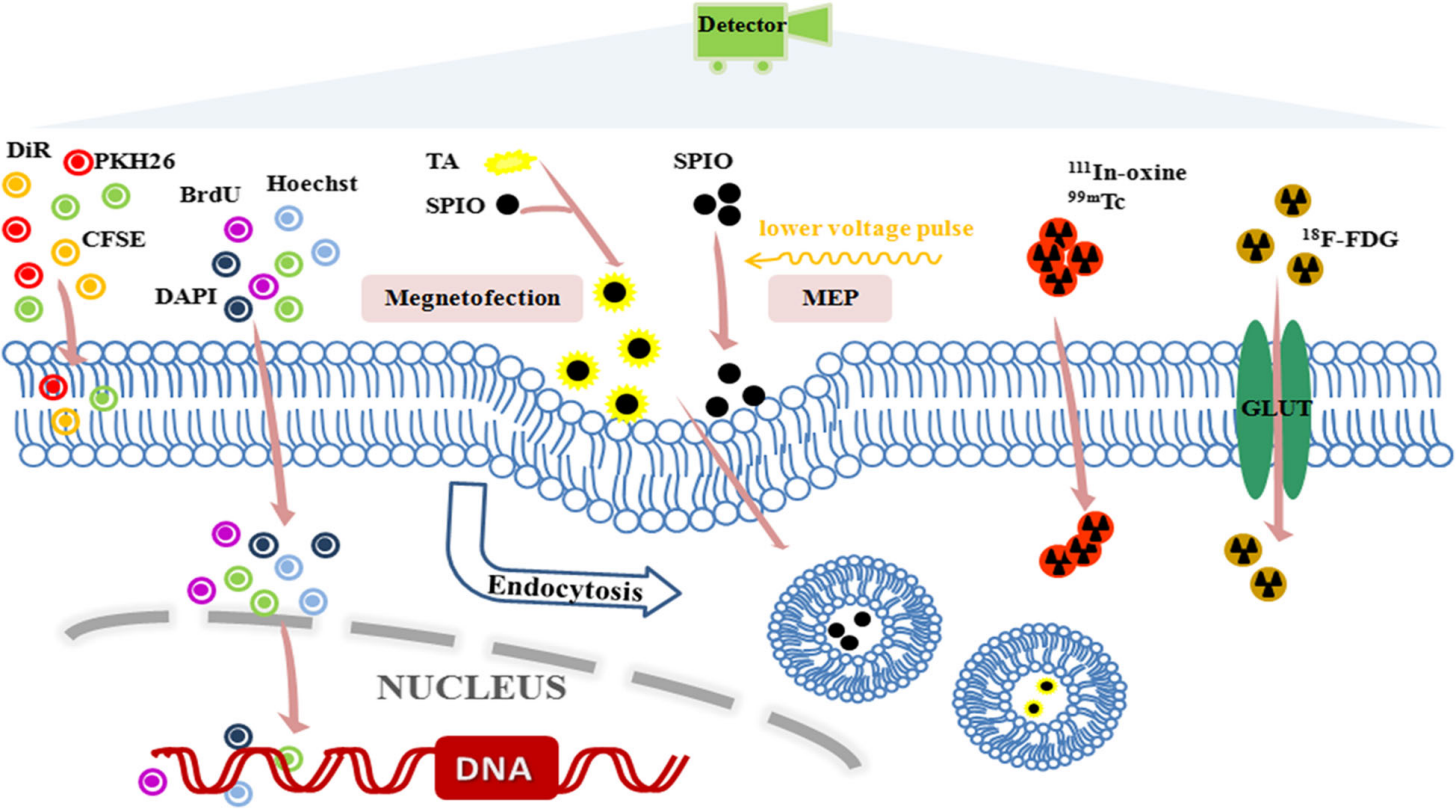
Schematic of direct labeling techniques for intrahepatic stem cell tracing. Stem cells are incubated with different tracer agents that enter the cells through various mechanisms, such as passive transport (fluorescent dyes, ^111^In-oxine, ^99m^Tc), endocytosis (SIPO), and uptake transporter (^18^F-FDG). Fluorescent dyes can respectively bind to the cell nucleus, membrane, or cytoplasm. Magnetofection and MEP are two methods for intracytoplasmatic magnetic labeling using SPIO. Tagged stem cells can be detected by imaging equipments, such as fluorescent microscope, MRI, PET, or SPECT. *TA* transfection agent, *SPIO* superparamagnetic iron oxides, ^*18*^ *F-FDG* ^18^F-fluorodeoxyglucose, *MEP* magnetoelectroporation, *DAPI* 4',6-diamidino-2-phenylindole, *BrdU* 5-bromo-2'-deoxyuridine, *DiR* 1,1-dioctadecyl-3,3,3,3-tetramethylindotricarbocyanine iodide, *CFSE* carboxyfluorescein succinimidyl amino ester

**Fig. 2:**
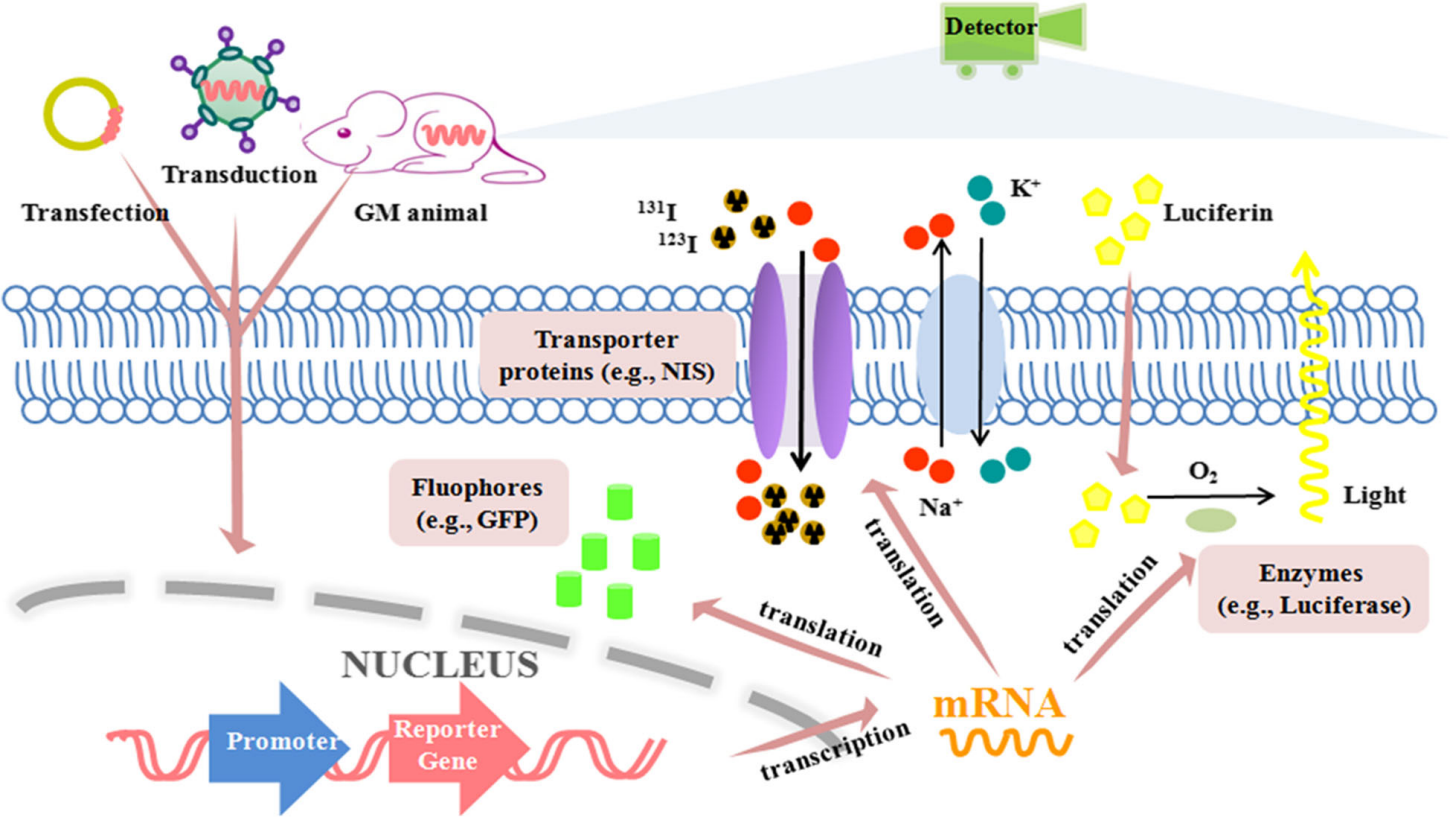
Schematic of reporter gene labeling for intrahepatic stem cell tracing. The reporter genes are transferred into the genome of target stem cells via transfection, transduction with viral vectors, or acquisition from GM animals. Activated by promoters, reporter genes encode various reporter proteins, such as fluorophores (e.g., GFP), enzymes (e.g., luciferase), transporter proteins (e.g., NIS), and so forth. Generated signals are captured by different imaging devices; for example, optical charge-coupled device, SPECT, or PET. *GM* genetically modified, *GFP* green fluorescent protein, *NIS* sodium iodide symporter

To date, quite a few clinical trials of stem cell-based therapies have been performed focusing on liver regeneration [60, 61]. However, most cases are in the early phase I/II stage. Improved liver function has been found to be small in some studies, and an average of only 55 % patients showed amelioration in histological tissues [62]. There is still a long way to go until the widespread application of stem cell therapies in clinical practice, which prompts us to translate from bedside to bench to furnish deeper insights into the fate of stem cells and treatment mechanisms. Imaging processes using direct labeling strategies and reporter genes enable us to elucidate key issues, including cellular survival, migration, biodistribution, and hepatogenic differentiation. The existing imaging strategies differ mainly in terms of depth penetration, spatial and temporal resolution, sensitivity, quantitative degree, molecular probes, and cost of imaging modalities. Every tracing technique has its inherent advantages and shortcomings owing to different mechanisms of action (Figs. 1 and 2). MRI is excellent for spatial resolution and soft-tissue contrast, but is far less sensitive than fluorescent imaging (10^–9^–10^–12^ mol/l) or radionuclide imaging (10^–11^–10^–12^ mol/l) [18]. Optical imaging allows for multilabeling and high-efficiency tracking, but is limited by unstable photobleaching and weak tissue penetration. The persistent expression of reporter genes is fit for long-term tracing. Nevertheless, concerns about the ionizing radiations from radioisotopes, leakage or diffusion of tracers, and immunogenic and ethical issues with reporter genes still require attention. A qualitative outline comparing some general features of these imaging modalities is presented in Table 4. The ideal imaging method for each study must be determined in light of the high sensitivity, resolution, and tracing time.

**Table 4:** Comparison of characteristics of imaging modalities available for intrahepatic stem cell tracing

Imaging technique	Probes	Imaging methods	Sensitivity	Spatial resolution	Temporal resolution	Penetration depth	Quantitative degree	Cost
Fluorescence imaging	Fluorophores, QDs, GFP/RFP	Direct labeling/reporter genes	+++	+	++	+	+to++	$
Bioluminescence imaging	Luciferin	Reporter genes	+++	+	++	+	+to++	$
MRI	SPIO, gadolinium	Direct labeling	+	+++	+	+++	++	
SPECT	^111^In, ^99m^Tc, ^131^I	Direct labeling/reporter genes	++	++	+	+++	++	$$
PET	^18^F, ^124^I	Direct labeling/reporter genes	++	++	+	+++	+++	$$$

+ common, ++ good, +++ excellent; $ cheap, $$ expensive, $$$ very expensive

*QD* quantum dot, *GFP* green fluorescent protein, *RFP* red fluorescent protein, *MRI* magnetic resonance imaging, *SPIO* superparamagnetic iron oxides, *SPECT* single photon emission computed tomography *PET* positron emission tomography

Noninvasive in-vivo imaging of stem cells stands at a critical point during the transition of treatment from bench to bedside. Clinical barriers will remain until the optimal cell types, timing of cellular graft, therapeutic dose, delivery route, and choices of endpoints are addressed. Therefore, it is vital to implement more large-scale clinical studies combined with imaging strategies to explore the trail of cell tracking in patients with liver diseases in the near future. Novel imaging strategies or combining imaging modalities might help to shed new light on the biological behavior of stem cells and the therapeutic mechanisms by which transplanted stem cells improve hepatic function and promote self-rehabilitation.

## Abbreviations

AFP: Alpha fetal protein
BLI: Bioluminescence imaging
BMSC: Bone marrow-derived mesenchymal stem cell
CCD: Charge-coupled device
CPN: Conjugated polymer-based water-dispersible nanoparticle
FISH: Fluorescence in-situ hybridization
FLI: Fluorescent imaging
GFP: Green fluorescent protein
HSV-tk: Herpes simplex virus type 1 thymidine kinase
iPSC: Induced pluripotent stem cell
LacZ: Galactosidase genes
MEP: Magnetoelectroporation
MNP: Fluorescent magnetic nanoparticle
MRI: Magnetic resonance imaging
NIS: Sodium iodide symporter
OLT: Orthotopic liver transplant
PET: Positron emission tomography
QD: Quantum dot
SPECT: Single-photon emission computed tomography
SPIO: Superparamagnetic iron oxides
